# Microbleeds show a characteristic distribution in cerebral fat embolism

**DOI:** 10.1186/s13244-021-00988-6

**Published:** 2021-03-31

**Authors:** Omar Giyab, Bendegúz Balogh, Péter Bogner, Orsi Gergely, Arnold Tóth

**Affiliations:** 1 Department of Medical Imaging, University of Pécs Medical School, Ifjúság út 13, 7624 Pécs, Hungary; 2 Department of Neurosurgery, University of Pécs Medical School, Rét utca 2, Pécs, 7623 Hungary; 3 MTA-PTE Clinical Neuroscience MR Research Group, University of Pécs Medical School, Ifjuság út 20, Pécs, 7624 Hungary

**Keywords:** Fat embolism, Cerebral fat embolism, Microbleed, DWI, SWI

## Abstract

This systematic review aims to test the hypothesis that microbleeds detected by MRI are common and show a characteristic pattern in cerebral fat embolism (CFE). Eighty-four papers involving 140 CFE patients were eligible for this review based on a systematic literature search up to 31 January 2020. An additional case was added from hospital records. Patient data were individually scrutinised to extract epidemiological, clinical and imaging variables. Characteristic CFE microbleed pattern resembling a “walnut kernel” was defined as punctuate hypointensities of monotonous size, diffusely located in the subcortical white matter, the internal capsule and the corpus callosum, with mostly spared corona radiata and non-subcortical centrum semiovale, detected by susceptibility- or T2* weighted imaging. The presence rate of this pattern and other, previously described MRI markers of CFE such as the starfield pattern and further diffusion abnormalities were recorded and statistically compared. The presence rate of microbleeds of any pattern, the “walnut kernel microbleed pattern”, diffusion abnormality of any pattern, the starfield pattern, and cytotoxic edema in the corpus callosum was found to be 98.11%, 89.74%, 97.64%, 68.5%, and 77.27% respectively. The presence rate between the walnut kernel and the starfield pattern was significantly (*p* < 0.05) different. Microbleeds are common and mostly occur in a characteristic pattern resembling a “walnut kernel” in the CFE MRI literature. Microbleeds of this pattern in SWI or T2* MRI, along with the starfield pattern in diffusion imaging appear to be the most important imaging markers of CFE and may aid the diagnosis in clinically equivocal cases.

## Key points

- Microbleeds detected by susceptibility- or T2* weighted imaging show a characteristic distribution in cerebral fat embolism.
- Such microbleed pattern may be just as commonly and more constantly present in cerebral fat embolism than the well-known “starfield pattern” detected by diffusion weighted imaging.
- Confluent cytotoxic edema is most commonly present in the corpus callosum.

## Introduction

Cerebral fat embolism (CFE) is part of the fat embolism syndrome (FES), which results from the intravascular embolisation of fat globules. FES is most commonly caused by displaced long bone fractures, or during orthopaedic procedures but it may also be caused by other conditions like sickle cell disease, severe pancreatitis, or following liposuction [1, 2].

First described by Zenker in 1862 the exact mechanism of CFE remains poorly understood. Both mechanical and biochemical theories have been proposed to explain the syndrome [3, 4]. The mechanical obstruction of arterial circulation by neutral fat globules, followed by a delayed biochemical toxic injury caused by free fatty acids is the most probable mechanism of tissue injury [5, 6]. CFE can occur without a patent foramen ovale, as small deformable fat globules can pass through the capillary circulation of the lung [7].

Cerebral fat embolism syndrome (CFES) occurs in up to 60% of patients with FES and is usually considered to be a self-limiting entity, but recent studies have also shown possible links to long term neurocognitive impairment [8–10]. The clinical presentation of CFES may vary considerably, symptoms typically appear between 12 to 72 h after the injury and may range from mild neurological impairment to alarming symptoms like coma or even death in the most severe cases [11].

There are no definitive clinical diagnostic tests and criteria developed, making the diagnosis of FES difficult [12, 13]. The diagnosis is usually based on a combination of symptoms, laboratory and imaging findings [9]. Currently the most commonly used diagnostic criteria have been proposed by Gurd and Wilson presented in Table 1 [14]. In this system, at least two positive major criteria, or one positive major and four positive minor criteria are considered suggestive of FES [14].

**Table 1:** Gurd and Wilson’s criteria

Gurd and Wilsion’s criteria*	
Major criteria	Minor criteria
Petechial rash	Tachycardia (> 110 bpm)
Respiratory insufficiency	Fever (> 38.5 °C)
Cerebral symptoms in non-head injury patients	Retinal changes (fat or petechiae)
	Jaundice
	Renal changes (oliguria, anuria, or lipiduria)
	Acute onset thrombocytopenia
	Acute drop in haemoglobin
	Elevated erythrocyte sedimentation rate
	Fat macroglobinemia

*The diagnosis of FES requires 2 major or 1 major and 4 minor criteria to be fulfilled

Many patients, however, do not develop the classic triad of cutaneous, respiratory and neurological symptoms [9]. Accordingly, the Gurd and Wilson’s criteria are not fulfilled, and the sole or first manifestation of FES may be neurologic, thus resulting in a diagnostic challenge to clinicians [9, 15]. Post-mortem pathological studies have shown CFE to be more common than previously thought indicating that it is underdiagnosed, and the clinical criteria used to diagnose the condition are not entirely reliable [16].

CT scans performed are typically without any specific finding, however may show widespread interval low density changes which should raise the suspicion of CFE [11]. Compared to CT, imaging findings in MRI are much more common in CFE [17]. The “starfield pattern” has been widely used in literature for describing the reversible imaging pattern of multifocal punctate lesions showing diffusion restriction. Other reported features include confluent cytotoxic edema in the white matter, and vasogenic lesions that may enhance [1, 11, 17]. Additional features of CFE that has been increasingly reported more recently are cerebral and cerebellar microbleeds depicted by susceptibility weighted imaging (SWI) or T2* MRI [18]. These studies and a case that we present in this review have led us hypothesize that microbleeds show a characteristic pattern in CFE. We aimed to test this hypothesis by a systematic review of the literature.

## Methods

### Literature search

We have searched the PubMed database up to 31 January 2020 (no time constraints were set for the earliest publication) for papers which included detailed imaging description or images of cases with CFE. We used the keywords “MR” or “MRI” and”fat emboli” or”fat embolism”, or “starfield pattern”. References of the included papers were then individually checked for additional articles. We also searched the PACS, and the hospital information system (HIS) of the University of Pécs for CFE cases. We used the ICD-10-CM diagnosis code T79.10 (traumatic fat embolism) for searching the PACS (MedView – ASPYRA LLC 7400 Baymeadows Way, Suite 101 Jacksonville, FL 32,256), and HIS (eMedSolution—T-Systems Magyarország Zrt. H-1097 Budapest, Könyves Kálmán krt. 36.) of the University of Pécs from 1 October 2016 (installation of MR scanner) until 31 January 2020. We have found one potentially suitable case for inclusion in this systematic review, whose legal representative was contacted and a signed informed consent was acquired for the anonymized inclusion and publication in this review. Search results were saved and organised via the commercially available Mendeley software (V1.19.5) and its web importer plug-in.

The exclusion criteria included recorded head trauma, poor image quality, unavailable images and poor image description, non-English language articles, animal experiment articles, CFE articles related to facial lipid injection procedure, and articles that are not available for online access.

### Data extraction

All included articles were individually scrutinised by a board certified neuroradiologist (O.G.) and other two board certified radiologists with a minimum of five-year experience in interpreting neuroradiological examinations (A.T. and P.B.) to collect relevant data for this review. Data were populated into an Excel (ver. 1908, Microsoft, Redmond, Washington, US) table for statistical evaluation.

If more than one case was presented in an article, then all the available information from each case was recorded separately. If only mean values were reported for a given variable regarding patients, then these data were not included in further evaluation. The time point of CT and MRI scans were recorded in days, and time point 0 was considered to be the time of arrival to the hospital. If mentioned, the Glasgow coma scale (GCS) was used to record the neurological status. Gurd and Wilson’s, and Schönfeld’s criteria were only recorded if they were mentioned in the article.

The extracted clinical and epidemiological variables and available patients, are presented in Table 2.

**Table 2:** Epidemiological and clinical data

Variable		Number (*n*)	Total* (Σ)	Rate (%)	SD**	Mean
*Epidemiological and clinical data*						
Patients included		141	141			
Male		72	141	71		
Female		29	141	29		
Age	Overall	101	141	71.63	18.5	42.1
	Males	72	101	71.29	20.1	36
	Females	29	101	28.71	24.2	58
Etiology	Fracture	79	141	56.03		
	Single bone fracture	38	141	26.95		
	Multiple bone fracture	37	141	26.24		
	Femur fracture	41	141	29.08		
	Tibia fracture	21	141	14.89		
	Polytrauma	27	141	19.15		
	Sickle cell disease	10	141	7.09		
Patent foramen ovale		8	141	5.67		
Initial neurological status (GCS)	15	76	85	89.41		
	14	4	85	4.71		
	< 14	5	85	5.88		
Gurd and Wilson’s criteria applied		43	141	30.49		
Time to first MRI in days		60	60		3.478	3.45
Follow up MRI performed		59	141	41.84		
Mortality		4	141	2.8		

*Total number of cases eligible for analysis

**Standard deviation

Regarding imaging findings, the presented images, text and supplementary materials were evaluated as well. Imaging findings were recorded if they were identifiable on the presented images, or clearly described in the text. Findings that were not identifiable in the presented images or were not mentioned in the text were considered to be absent. In case of any discrepancy between the text and the available images, they were double-checked by each participating radiologist, and were excluded from the statistical analyses if the discrepancy was confirmed by all readers. Ambiguous imaging findings (due to poor image quality, or partially visible pathology) if not detailed in the text were not considered in the statistical calculations.

The hypothesized, CFE characteristic microbleed pattern was defined as the diffuse presence of round microbleeds (punctate focal hypointensities) of monotonous size in the subcortical white matter (involving but not limited to the U-fibers), internal capsule and the corpus callosum, mostly sparing the corona radiata and the non-subcortical centrum semiovale on T2* GRE or SWI images. As authors believe this pattern resembling the appearance of a walnut kernel, it is further referred to as the “walnut kernel pattern”.

The starfield pattern as described by Parizel et al. refers to the presence of scattered bright spots in a dark background in DWI with diffusion restriction, was considered positive if it was identifiable in the presented DWI and ADC images, or was clearly described in text [1]. Cases referred to as starfield positive without presented ADC or clear reference to diffusion restriction, but with very typical starfield pattern in the presented DWI agreed by the three reviewing radiologists in the present study were also accepted as definitive starfield pattern. We categorised a case as starfield negative if there was no mention or image of the starfield pattern, ADC map did not show restricted diffusion, and there was no additional mention of restricted diffusion in the text.

Confluent diffusion restriction in the corpus callosum, if present, was also recorded.

Further recorded imaging findings included confluent cytotoxic edema in the white matter (other than corpus callosum), vasogenic edema in the white matter, petechial hemorrhages of non-characteristic pattern, and chronic sequel – atrophy.

### Data analysis

Descriptive statistics were used to summarise the recorded parameters. To assess the presence rate of "walnut kernel microbleed pattern" for CFE, the number of characteristic CFE pattern cases was divided by the number of all cases where it was possible to evaluate the presence of microbleed pattern. Similarly, presence rate of starfield pattern for CFE was calculated by dividing the number of cases with definitive starfield pattern by all cases in which it was possible to definitively evaluate. Characteristic microbleed pattern and starfield pattern presence rates were compared using Fisher exact test. For a more direct, one-sample comparison, the presence rates were compared by McNemar test in a subset of patients for whom it was possible to evaluate both the microbleed and the starfield patterns. Both Fisher and McNemar tests were run in MedCalc Statistical Software version 18.11.3 (MedCalc Software bvba https://www.medcalc.org; 2019, [19]) and tests were regarded significant if yielding a p value less than 0.05.

To present temporal features of the walnut kernel microbleed pattern and the starfield pattern, the included cases were assigned into three stages: acute stage (first 4 days of hospitalisation), subacute stage (5–14 days) and late stage (14 + days). In all stages, four groups were created: starfield pattern positive and negative groups and walnut kernel pattern positive and negative groups. For this analysis, we disqualified case studies and reviews if the exact time point of the MRI for each patient was not reported.

## Results

**Fig. 1:**
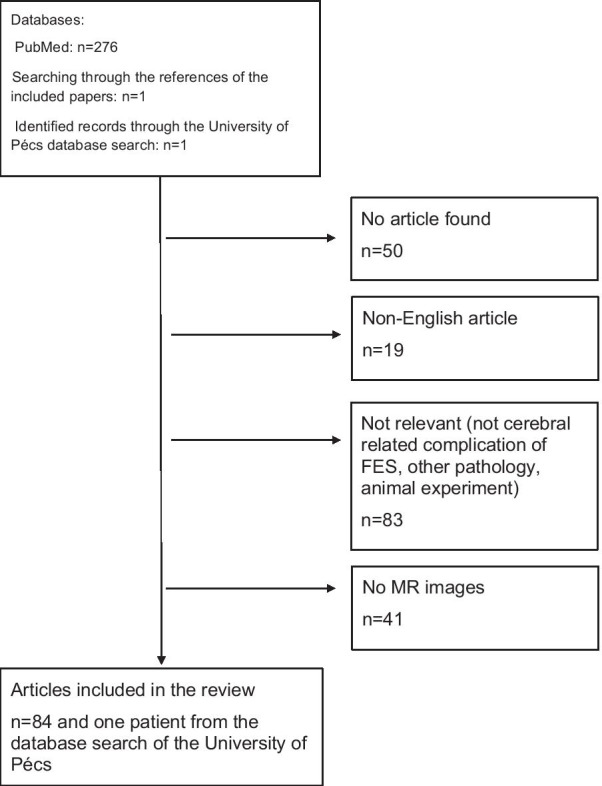
Quality of Reporting of Meta-analysis standards (QUOROM) flow diagram of articles included in this systematic review

**Fig. 2:**
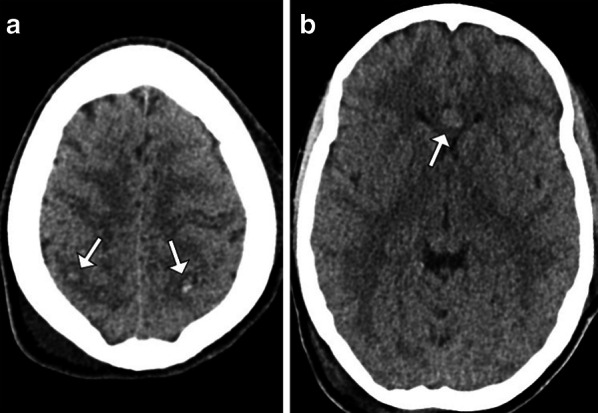
A case showing microhemorrhages as seen on CT. Axial native CT scan images at the level of the centrum semiovale and the basal ganglia of a 16-year-old polytraumatised female patient acquired on postoperative day 11 after hospital admission. Multiple subcortical punctiform hyperdensities presumably representing microhemorrhages are seen bilaterally in the subcortical white matter (**a**), and a larger hyperdensity is seen in the genu of the corpus callosum (**b**)

**Fig. 3:**
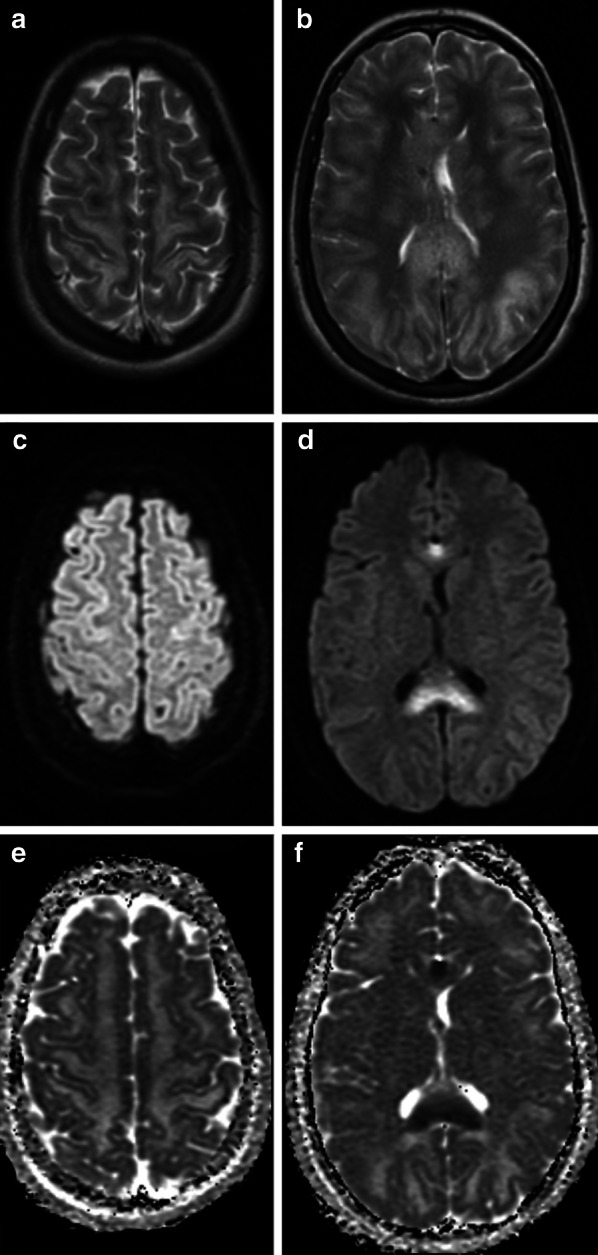
A case representing the various patterns of diffusion abnormalities seen in CFE. T2 (**a**, **b**), DWI (**c**, **d**), and ADC (**e**, **f**) MRI images at the level of the centrum semiovale (left column), and at the level of the basal ganglia (right column) of a 16-year-old polytraumatised female patient acquired on day 12 after hospital admission. T2 weighted images show hyperintense signal in the subcortical white mater, internal capsule and the corpus callosum (**a**, **b**). Diffusion-weighted MR Image with a b value of 1000 s/mm^2^ (**c**, **d**), and ADC (**e**, **f**) images show cytotoxic edema affecting the corpus callosum and facilitated diffusion over the subcortical white matter

**Fig. 4:**
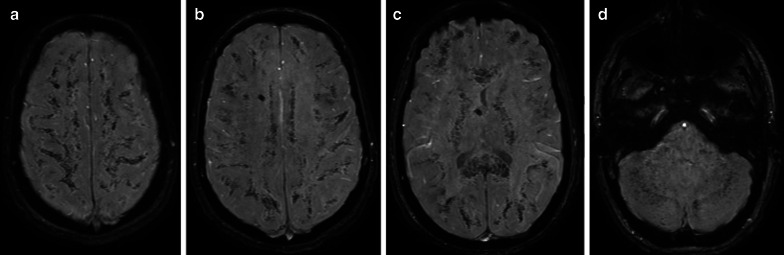
A case representing the walnut kernel microbleed pattern. SWI (**a**–**d**) MRI images at the level of the centrum semiovale (**a**), at the level of the corona radiata (**b**), at the level of the basal ganglia (**c**), and at the level of the brainstem and the cerebellum (**d**) of a 16-year-old polytraumatised female patient acquired on day 12 after hospital admission. SWI shows very high number of monotonous punctuate microbleeds in the subcortical white matter, the internal capsule, the corpus callosum, the cerebellum and the brainstem resembling a “walnut kernel”. The larger hypointensity visible in the right centrum semiovale and near the third ventricle is due to a ventricular drain (**a**–**c**)

**Fig. 5:**
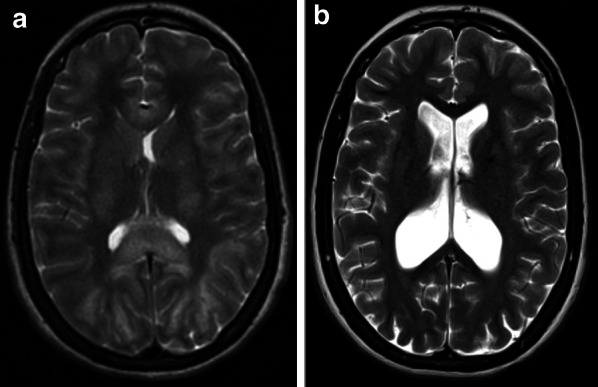
A case representing the long-term radiological consequences of CFE. T2 MRI images at the level of the basal ganglia of a 16-year-old polytraumatised female patient acquired on day 12 after hospital admission (**a**), and 16 months later (**b**). The long-term follow-up images show resolution of the subcortical and corpus callosum T2 hyperintensities, and dilation of the intergyral sulci, and the lateral ventricles indicative of cerebral atrophy (**b**)

The literature search identified 277 articles, from which 193 articles were excluded, resulting in a total of 84 included articles, for details see Fig. 1 flow chart. The oldest included paper was published in 1998 [15]. In our institution’s database search, we have found one case that was tagged by the ICD-10-CM diagnosis code T79.10 for traumatic fat embolization. This was the case of a previously healthy 16-year-old female patient who was run over by a car and suffered severe thoracic and abdominal injuries including right sided serial rib fractures, right sided hemothorax, bilateral pneumothorax, diaphragmatic and hepatic rupture. No direct head injury was recorded based on clinical evaluation and the negative admission CT. Due to delayed awakening on the sixth postoperative day, a head CT was performed which showed multiple punctiform hyperdensities which disappeared by day 17 (Fig. 2.). The typical starfield pattern of CFE, were not seen on the MR images that were acquired on day 12 after admission to the hospital, but there was facilitated diffusion in the subcortical white matter, and diffusion restriction was limited to the corpus callosum only (Fig. 3.). The case showed the walnut kernel microbleed pattern in SWI, surrounded by extracellular edema, except for the corpus callosum where cytotoxic edema was shown (Figs. 3, 4). Considering the results of lab tests and previous reports, the diagnosis of cerebral fat embolism was made. Following supportive care and three months long rehabilitation, a near-complete neuropsychological recovery was achieved. Long term follow-up MR images 16 months later revealed signs of cerebral atrophy (Fig. 5.).

From the 84 included articles, there were 73 case reports, 6 review papers, 4 original papers, and 1 review pictorial essay. 140 patients from the included articles satisfied our inclusion criteria and were included in this review, in addition to the previously mentioned case from our own databases, resulting in an overall patient number of 141.

The extracted epidemiological and clinical data are summarised in Table 2.

In the included literature SWI was done in 40 cases, T2* was performed in 21 cases, and both SWI and T2* measurements were performed in 12 cases. Microbleeds in general, were present in 52 out of 53 cases (98.11%). The "walnut kernel microbleed pattern" was found in 35 out of 39 evaluable cases resulting in a presence rate of 89.74%.

**Fig. 6:**
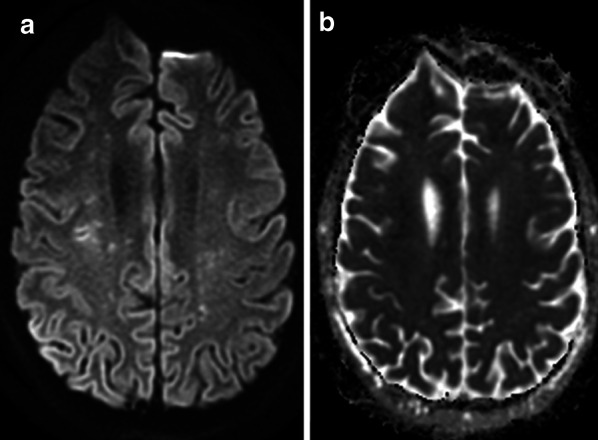
A case representing the starfield pattern of restricted diffusion. MRI scan of an 18-year-old man with a closed displaced fracture of the left femoral shaft after a high velocity motor vehicle accident. Diffusion-weighted MR Image (10000/89; b value, 1000 s/mm^2^) showing foci of hyperintensities within both centrum ovale (**a**), and the corresponding ADC map confirming restricted diffusion (**b**). Published under the permission of G. Bierry and S. Kremer, Department of Radiology, University Hospital of Strasbourg, Strasbourg, France

Diffusion abnormality in general, was seen in 97.64% of the cases (*n* = 124). The starfield pattern description was used in 70.08% (*n* = 89) of the cases, but only 19.10% (*n* = 17) of these cases were confirmed with an ADC map (Fig. 6.). The majority of the presented cases relied on T2 or DWI images only for using the starfield pattern term. However, in 59 out of these cases, diffusion restriction was referred to in the text, in other 8 cases the authors did not use the term starfield pattern, yet it was clearly present in the shown images, and in 3 cases the authors described the lesions as starfield positive, but there was no further accurate detailing of this finding presented. Thus, definitive starfield pattern was ascertained in 87 out of 127 cases yielding a presence rate of 68.5%. In 3.3% (*n* = 4) the term was used to describe foci of vasogenic edema, large confluent areas of cytotoxic edema, or infarcts were described on T2 images alone [20–23]. Confluent restricted diffusion was seen in the corpus callosum in 77.27% (34 out of 44 cases). On follow-up studies brain atrophy has been confirmed in 8 cases [1, 17, 24–29].

CFE related imaging findings are summarised in Table 3.

**Table 3:** Radiological findings

Variable	(*n*)	(Σ)*	Rate (%)	References
Radiological findings				
Microbleeds	52	53^a^	98.11	[17, 18, 20, 21, 24–27, 39–71]
Walnut kernel microbleed pattern**	35	39^b^	89.74	[17, 18, 23–26, 39, 41, 45–47, 49, 50, 52–54, 56, 58, 61–63, 66, 70–73]
Diffusion abnormality	124	127^c^	97.64	[1, 8, 9, 11, 17, 18, 22–30, 39–55, 57–59, 61–71, 73–104]
Definitive starfield pattern**	87	127^c^	68.5	[1, 9, 11, 17, 18, 23, 24, 26–28, 40–42, 44, 46, 48, 50–52, 54, 58, 59, 61–65, 67–69, 74–78, 80–85, 88, 90, 91, 93–96, 98, 99]
Confluent cytotoxic edema in white matter	41	82^d^	50	[8, 17, 18, 24–26, 28, 29, 42, 44, 45, 47, 50–52, 54, 55, 65, 70, 76, 78, 84–87, 89, 90, 92–94, 97, 98, 100, 103–105]
Cytotoxic edema in the corpus callosum	34	44^e^	77.27	[9, 17, 18, 23–26, 28, 40, 44, 45, 61, 62, 65, 66, 70, 72, 81, 83–85, 88, 89, 93, 94, 96–98, 101]
Vasogenic edema lesions	17	58^f^	29.31	[8, 11, 17, 23, 26, 29, 30, 41, 42, 47, 50, 52, 55, 56, 78, 89, 92]
Atrophy	9	9^g^	100	[1, 17, 24–29]

*Total number of cases eligible for analysis

**For definition see methods

^a^Cases with presented susceptibility- or T2* images, or with no presented images but with clear description regarding microbleed presence

^b^Cases with susceptibility- or T2* images in which the subcortical white matter, internal capsule, and the corpus callosum were evaluable, and cases with no presented images but description of findings regarding microbleeds in the specified locations

^c^Cases with presented DWI, or DWI and ADC. Cases with no such presented imaging but with clear description of any diffusion abnormality, or the absence of any diffusion abnormality were also included

^d^Cases with presented DWI and ADC images. Cases without presented images but with description of findings regarding the presence or absence of confluent cytotoxic edema were also included

^e^Cases with DWI and ADC images where the corpus callosum is visible. Cases without presented images but with clear description regarding the presence or absence of corpus callosum diffusion restriction were also included

^f^Cases with presented DWI and ADC images. Cases without presented images but with clear description regarding the presence or absence of lesions of facilitated diffusion were also included.

^g^Cases with presented follow-up MR or CT images. Cases without presented images but with clear description regarding the presence or absence of atrophy in the late stage

Between the presence rates of the walnut kernel and definitive starfield patterns (89.74% vs. 68.5%), Fisher exact test showed a significant (*p* = 0.0073) difference. In the subset of patients where both microbleed and starfield patterns were possible to be evaluated (*n* = 29), the "walnut kernel pattern" was present in 27 cases (93.1%), while the starfield pattern was present in 12 cases (41.38%). The McNemar test showed these rates to be significantly different (*p* = 0.0003).

**Fig. 7:**
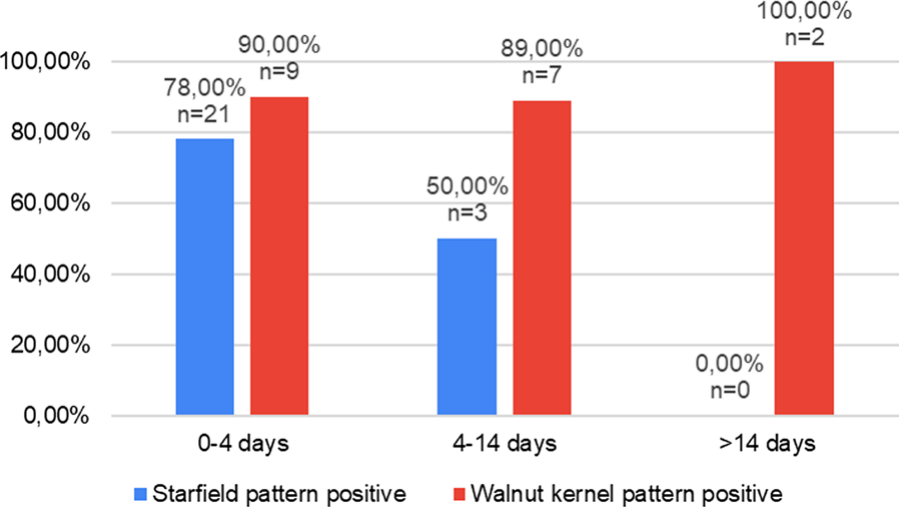
Bar graph indicating the positive proportion of radiological patterns against time. The starfield pattern was mostly present within the first 4 days after injury, while walnut kernel microbleed pattern had a more consistent presence among the different time periods

Regarding the temporal characteristics of the lesions (Fig. 7.), the starfield pattern was mostly present within the first 4 days after injury where 21 positive and 6 negative cases existed, while only 3 positive cases were reported along with 3 negative cases in the 4–14 days period. In turn, the walnut kernel microbleed pattern had a more consistent presence among time periods, with a case count of 9 positive vs 1 negative until day 4, 7 positive vs 1 negative in the 4–14 day period and 2 positive in the day 14 + period.

## Discussion

Since the clinical diagnosis of CFE is often difficult, imaging could provide significant help. We hypothesized that the increasingly described microbleeds and their particular distribution, the walnut kernel pattern (diffuse uniform punctiform microbleeds located in the subcortical region, the corpus callosum, and in the internal capsule) maybe an important marker for CFE.

Reviewing the literature and our database, it can be noted that although the authors have previously not referred to this actual pattern of microbleeds, the walnut kernel pattern appears to be indeed very common in CFE, as it was present in 89.74% of the cases. Recognizing this specific pattern could be utmost important because microbleeds in other distributions can be associated with other, possibly coinciding pathologies such as diffuse axonal injury (DAI) or sepsis, but they are also common in cardiovascular diseases.

In contrast to the walnut kernel pattern, microbleeds in DAI are seen in groups with varying size and shape (linear, curvilinear, to ovoid), in specific locations [18]. Hypertension associated microbleeds are typically seen only in deeper areas of the brain, while in amyloid angiopathy the microbleeds show a diffuse lobar pattern of more heterogeneous, mainly larger size [30].

Acute haemorrhagic leucoencephalitis is generally also associated with widespread white matter T2 lesions, the microbleeds are more often randomly distributed, and its clinical presentation is substantially different [31].

Diffuse cerebral microbleeds have been reported in up to 2% of patients receiving extracorporeal membrane oxygenation (ECMO) [33]. While having a similarly favourable prognosis to CFE in the published cases, diffuse cerebral microbleeds related to ECMO also have a quite similar distribution, therefore it is very important for radiologist to be informed about the patient undergoing such therapy [34, 35]. Disseminated cerebral microhemorrhages have been described as a late complication in critically ill COVID-19 patients as well [36]. When compared to CFE, microbleeds in critically ill patients such as in sepsis, disseminated intravascular coagulopathy, COVID-19 infection, and patients undergoing ECMO therapy tend to be more variable in size, and shape [32, 36–38]. To date, the exact pathomechanism of cerebral microbleeds in the critically-ill in general and in patients undergoing ECMO therapy is not known, but a commonly accepted speculation is that it is probably multifactorial in its nature, involving an ischemic and/or a biochemical insult induced a thrombotic or embolic event, hence the authors speculate that the underlying pathomechanism could be similar to that of CFE. Further imaging research and accompanying post-mortem studies might elucidate differential diagnosis and the exact mechanisms of microbleeds in critically ill patients.

Beyond microbleeds several types of CFE MR signs have been described in previous studies. According to a comprehensive review published in 2014, the imaging patterns of CFE were classified in to 4 groups (scattered cytotoxic edema, confluent cytotoxic edema in white matter, vasogenic edema lesions that may enhance, petechial hemorrhage of white matter, and chronic sequel), amongst which the starfield appearance was the most commonly encountered (61.5% of 44 MRI scans) [17]. In our review, it was possible to include more recent papers and therefore a larger number of patients were analysed that revealed a very similar occurrence rate 68.5% of the starfield pattern in CFE. However, the more recent papers included also considerably more SWI and T2* studies, revealing that microbleeds, and specifically the walnut kernel pattern may be even more common than the starfield pattern.

An emerging concern regarding the starfield pattern seems to be that there is a degree of uncertainty around its use in the literature. Originally, Parizel et al. has described the starfield pattern as “scattered bright spots on a dark background” on DW-MRI, which, according to their interpretation, “presumably reflect foci of cytotoxic edema” according to low ADC values [1]. Since then this expression has been used widely in the literature, but often without considering the ADC values. According to our findings this was the case in around half of the reviewed patients. In a few published cases the starfield pattern was used to describe confluent T2 hyperintense white matter lesions potentially representing other pathologies like vasogenic edema, while in other reports lesions of obviously high ADC values were referred to as starfield pattern.

From a practical point of view, it is important to note that compared to the starfield pattern, over time, microbleeds could be more consistently identified on MRI. Although due to the small number of corresponding cases, reasonable statistical analysis was not possible, it seems that the starfield pattern is typically present in the first 4 days, as there are much more positive than negative cases in this period, whereas in the 4–14 day time interval the number of positive and negative cases are equal. In contrast, irrespective of the time point, microbleeds were always more commonly present than absent—indicating constant visibility (Fig. 7). This is in line with the article published by Kuo et al. [17].

An additional remarkable observation of our review is that the previously described “confluent cytotoxic edema” is mostly present in the corpus callosum, with a prevalence of 77.27%, which is comparable to the starfield and walnut kernel patterns.

There is limited data in the literature regarding the chronic radiological sequel of CFE. Although the long term prognosis of CFE is generally considered to be good, cerebral atrophy and persistent T2 hyperintensities can be seen in some cases where follow-up imaging has been performed [1, 17, 24–29].

This study has certain limitations. First, comparison of the rates of the starfield and walnut kernel patterns is somewhat limited since considerably more articles investigated diffusion abnormalities than microbleeds. Second, the presence rates of the investigated imaging patterns do not necessarily reflect their true sensitivity, since it is possible that authors published their imaging results more likely in case of positive findings, causing seemingly higher presence rates of these imaging markers. Still, it can be safely postulated that the walnut kernel microbleed pattern, the starfield pattern and corpus callosum diffusion restriction are the most common imaging alterations in CFE. Third, data regarding chronic changes induced by CFE are scarce and therefore the prevalence of cerebral atrophy remains unknown.

## Conclusion

Microbleeds are very common and mostly occur in a characteristic pattern resembling a “walnut kernel” in the CFE MRI literature. Microbleeds of this pattern in SWI or T2* MRI, along with the starfield pattern and corpus callosum diffusion restriction in DWI/ADC appear to be the most important imaging markers of CFE and may aid the differential diagnosis in clinically equivocal cases.

## Data Availability

The datasets generated during the current study are available from the corresponding author upon request.

## Abbreviations

CFE: Cerebral fat embolism
CFES: Cerebral fat embolism syndrome
DAI: Diffuse axonal injury
ECMO: Extracorporeal membrane oxygenation
FES: Fat embolism syndrome
GCS: Glasgow coma scale
HIS: Hospital information system

## References

[1] Parizel PM, Demey HE, Veeckmans G (2001). Early diagnosis of cerebral fat embolism syndrome by diffusion-weighted MRI (starfield pattern). Stroke.

[2] Shaikh N (2009). Emergency management of fat embolism syndrome. J Emergencies, Trauma Shock.

[3] Beiträge zur normalen und pathologischen Anatomie der Lunge - Friedrich Albert Zenker -. In: Norm. und Patol. https://books.google.lu/books?id=4zpCAAAAcAAJ&printsec=frontcover&hl=de#v=onepage&q&f=false. Accessed 8 Feb 2020

[4] Müller C, Rahn BA, Pfister U, Meinig RP (1994). The incidence, pathogenesis, diagnosis, and treatment of fat embolism. Orthop Rev.

[5] Simon AD, Ulmer JL, Strottmann JM (2003). Contrast-enhanced MR imaging of cerebral fat embolism: case report and review of the literature. Am J Neuroradiol.

[6] Kamenar E (1980). Burger PC Cerebral fat embolism: a neuropathological study of a microembolic state. Stroke.

[7] Byrick RJ, Mullen JB, Mazer CD, Guest CB (1994). Transpulmonary systemic fat embolism: studies in mongrel dogs after cemented arthroplasty. Am J RespirCrit Care Med.

[8] Pfeffer G, Heran MKS (2010). Restricted diffusion and poor clinical outcome in cerebral fat embolism syndrome. Can J NeurolSci.

[9] Scarpino M, Lanzo G, Lolli F, Grippo A (2019). From the diagnosis to the therapeutic management: cerebral fat embolism, a clinical challenge. Int J Gen Med.

[10] Allen M (2016). Cerebral fat microembolism and its potential role in postoperative cognitive dysfunction after major orthopaedic surgery. J Bone JtSurg - Am.

[11] Dunkel J, Roth C, Erbguth F (2017). Cerebral fat embolism: clinical presentation, diagnostic steps and long-term follow-up. EurNeurol.

[12] Bollineni VR, Gelin G, Van Cauter S (2019). No Title. J Belgian SocRadiol.

[13] Kwiatt ME, Seamon MJ (2013). Symposium: embolism in the intensive care unit fat embolism syndrome. Int J CritIllnInjSci.

[14] Gurd AR, Wilson RI (1974). The fat embolism syndrome. J Bone Joint Surg Br.

[15] Bardana D, Rudan J, Cervenko F, Smith R (1998). Fat embolism syndrome in a patient demonstrating only neurologic symptoms. Can J Surg.

[16] Georgopoulos D, Bouros D (2003). Fat embolism syndrome: clinical examination is still the preferable diagnostic method. Chest.

[17] Kuo KH, Pan YJ, Lai YJ (2014). Dynamic MR imaging patterns of cerebral fat embolism: a systematic review with illustrative cases. Am J Neuroradiol.

[18] Rutman AM, Rapp EJ, Hippe DS (2017). T2∗-weighted and diffusion magnetic resonance imaging differentiation of cerebral fat embolism from diffuse axonal injury. J Comput Assist Tomogr.

[19] Schoonjans F, Zalata A, Depuydt CE, Comhaire FH (1995). MedCalc: a new computer program for medical statistics. Comput Methods Programs Biomed.

[20] Blitstein MK, Tung GA (2007). MRI of cerebral microhemorrhages. Am J Roentgenol.

[21] Rothberg DL, Makarewich CA (2019). Fat embolism and fat embolism syndrome. J Am AcadOrthopSurg.

[22] Il SuhS, Seol HY, Seo WK, Koh SB (2009). Cerebral fat embolism: susceptibility-weighted magnetic resonance imaging. Arch Neurol.

[23] Stienen MN, Gautschi OP (2012). Cerebral fat embolism after severe road traffic accident. Am J Emerg Med.

[24] Metting Z, Rödiger LA, Regtien JG, van der Naalt J (2009). Delayed coma in head injury: consider cerebral fat embolism. ClinNeurolNeurosurg.

[25] Lee J (2008). Gradient-echo MRI in defining the severity of cerebral fat embolism. J ClinNeurol.

[26] Hermann B, Brisson H, Langeron O (2018). Unexpected good outcome in severe cerebral fat embolism syndrome. Ann ClinTranslNeurol.

[27] De Feiter PW, Van Hooft MAA, Beets-Tan RG, Brink PRG (2007). Fat embolism syndrome: yes or no?. J Trauma - Inj Infect Crit Care.

[28] Guillevin R, Vallée JN, Demeret S (2005). Cerebral fat embolism: usefulness of magnetic resonance spectroscopy. Ann Neurol.

[29] Eguia P, Medina A, Garcia-Monco JC (2007). The value of diffusion-weighted MRI in the diagnosis of cerebral fat embolism. J Neuroimaging.

[30] Scarpino M, Lanzo G, Cappelli F (2016). Cerebral fat embolism after video-assisted thoracic surgery. Ann ThoracSurg.

[31] Acute hemorrhagic encephalomyelitis in childhood: Case report and literature review. https://www.ncbi.nlm.nih.gov/pmc/articles/PMC3173916/. Accessed 24 Dec 2020 10.4103/1817-1745.84408 PMC3173916 21977089

[32] Haller S, Vernooij MW, Kuijer JPA (2018). Cerebral microbleeds: imaging and clinical significance. Radiology.

[33] Luyt CE, Bréchot N, Demondion P (2016). Brain injury during venovenous extracorporeal membrane oxygenation. Intensive Care Med.

[34] Gijs J, Lambert J, Meyfroidt G, Demeestere J (2018). Cerebral microbleeds and intracerebral hemorrhage associated with veno-venous extracorporeal membrane oxygenation. ActaNeurolBelg.

[35] (2015) CORRESPONDENCE diffuse cerebral microbleeds after extracorporeal membrane oxygenation support 10.1164/rccm.201411-2118LE 25723825

[36] Radmanesh A, Derman A, Lui YW (2020). COVID-19–associated diffuse leukoencephalopathy and microhemorrhages. Radiology.

[37] Cannac O, Martinez-Almoyna L, Hraiech S (2020). Critical illness-associated cerebral microbleeds in COVID-19 acute respiratory distress syndrome. Neurology.

[38] Thurnher MM, Boban J, Röggla M, Staudinger T (2021). Distinct pattern of microsusceptibility changes on brain magnetic resonance imaging (MRI) in critically ill patients on mechanical ventilation/oxygenation. Neuroradiology.

[39] Akoh CC, Schick C, Otero J, Karam M (2014). Fat embolism syndrome after femur fracture fixation: a case report. Iowa Orthop J.

[40] Molière S, Kremer S, Bierry G (2018). Case 254: posttraumatic migrating fat embolus causing fat emboli syndrome. Radiology.

[41] Bodanapally UK, Shanmuganathan K, Saksobhavivat N (2013). MR imaging and differentiation of cerebral fat embolism syndrome from diffuse axonal injury: application of diffusion tensor imaging. Neuroradiology.

[42] Chen JJS, Ha JC, Mirvis SE (2008). MR imaging of the brain in fat embolism syndrome. EmergRadiol.

[43] Shobha N, Bermejo PG, Bhatia R (2011). Multimodal imaging tools for diagnosis of fat embolism. J Emerg Trauma Shock.

[44] Mittal MK, Burrus TM, Campeau NG (2013). Pearls & oy-sters: good recovery following cerebral fat embolization with paroxysmal hyperactivity syndrome. Neurology.

[45] Yeap P, Kanodia AK, Main G, Yong A (2015). Role of susceptibility-weighted imaging in demonstration of cerebral fat embolism. BMJ Case Rep.

[46] Fernández-Torre JL, Burgueño P, Ballesteros MA (2015). Super-refractory nonconvulsive status epilepticus secondary to fat embolism: a clinical, electrophysiological, and pathological study. Epilepsy Behav.

[47] Lützen N, Niesen WD, Kalbhenn J, Urbach H (2016). Teaching neuroimages: cerebral fat embolism after cemented hip replacement. ClinNeuroradiol.

[48] Burns JD, Noujaim D, Scott BJ (2017). Susceptibility-weighted imaging diagnosis of cerebral fat embolisfile. The Neurohospitalist.

[49] Huang L-C, Wu M-N, Chen C-H, Huang P (2013). Susceptibility-weighted imaging in patient with consciousness disturbance after traffic accident. Am J Emerg Med.

[50] Zaitsu Y, Terae S, Kudo K (2010). Susceptibility-weighted imaging of cerebral fat embolism. J Comput Assist Tomogr.

[51] Dhakal LP, Bourgeois K, Barrett KM, Freeman WD (2015). The “starfield” pattern of cerebral fat embolism from bone marrow necrosis in sickle cell crisis. The Neurohospitalist.

[52] Gupta B, Kaur M, Dsouza N (2011). cerebral fat embolism: a diagnostic challenge. Saudi J Anaesth.

[53] Huang P, Lin W-C, Huang P-K, Khor G-T (2009). Susceptibility weighted imaging in a patient with paroxysmal sympathetic storms. J Neurol.

[54] Zakhari N, Castillo M, Torres C (2016). Unusual cerebral emboli. Neuroimaging Clin N Am.

[55] Report C (2015) Concomitant fat embolism syndrome and pulmonary embolism in a patient with a femoral shaft fracture. 20–23. https://doi.org/10.1002/ams2.127 10.1002/ams2.127 PMC5667392 29123766

[56] Buskens CJ, Gratama JWC, Hogervorst M (2008). Encephalopathy and MRI abnormalities in fat embolism syndrome: a case report. Med Sci Monit.

[57] Yanagawa Y, Kaneko N, Sakamoto T, Okada Y (2007). Fat embolism syndrome with multiple hypointensity signals detected by head magnetic resonance imaging demonstrating a favorable outcome: a case report. Am J Emerg Med.

[58] Kang JH, Hargett CW, Sevilis T, Luedke M (2018). Sickle cell disease, fat embolism syndrome, and “starfield” pattern on MRI. NeurolClinPract.

[59] Liauw L, van Buchem MA, Feuth JD (1998). Cerebral fat embolism. EurRadiol.

[60] Skalski KA, Kessler AT, Bhatt AA (2018). Hemorrhagic and non-hemorrhagic causes of signal loss on susceptibility-weighted imaging. EmergRadiol.

[61] Lamotte G, Williams C (2017). Mystery case: a case of fulminant encephalopathy in a 69-year-old woman. Neurology.

[62] Scheifer C, Lionnet F, Bachmeyer C (2017). cerebral fat embolism in hemoglobin SC disease. Am J Med.

[63] Gibbs WN, Opatowsky MJ, Burton EC (2012). Airp best cases in radiologic-pathologic correlation: cerebral fat embolism syndrome in sickle cellβ-thalassemia. Radiographics.

[64] Mossa-Basha M, Izbudak I, Gurda GT, Aygun N (2012). Cerebral fat embolism syndrome in sickle cell anaemia/β-thalassemia: Importance of susceptibility-weighted MRI. ClinRadiol.

[65] Kokatnur L, Rudrappa M, Khasawneh KR (2015). Cerebral fat embolism: use of MR spectroscopy for accurate diagnosis. Ann Indian AcadNeurol.

[66] Alobeidi F, Inusa BPD, Singh RR, U-King-Im JM (2015). Cerebral microhaemorrhages secondary to fat embolus syndrome in sickle cell disease. Postgrad Med J.

[67] Bollineni VR, Gelin G, Van Cauter S (2019). Cerebral fat embolism syndrome. J Belgian SocRadiol.

[68] Chuang H-Y, Kung L-C, Huang M-Y (2018). An injured man with acute altered mental status. Emerg Med J.

[69] OliveraArencibia Y, Vo M, Kinaga J (2018). Fat embolism and nonconvulsive status epilepticus. Case Rep Neurol Med.

[70] Looby S, Royston D, Brett F (2017). Complicated fall in a 78-year-old lady. Brain Pathol.

[71] Loupy A, Laissy JP, Klein I (2008). Fat emboli unleashed: an exceptional etiology of encephalitis in sickle cell disease. Ann Hematol.

[72] Medina FJ, Marquez JC, Castillo M (2012). Cerebral fat embolism detection with susceptibility-weighted images in sickle cell disease. Neuroradiol J.

[73] Singh DR, Chawla A, Peh WC (2018) Clinics in diagnostic imaging (184). Fat embolism syndrome (FES). Singapore Med J 59:159–162. https://doi.org/10.11622/smedj.2018029 10.11622/smedj.2018029 PMC5861340 29568848

[74] Kammeyer R, Devnani R, Mehta R (2016). Cerebral fat embolism syndrome mimicking thrombotic thrombocytopenic purpura in a patient with hemoglobin SC disease. Am J Hematol.

[75] Shaikh N, Mahmood Z, Ghuori SI (2018). Correlation of clinical parameters with imaging findings to confirm the diagnosis of fat embolism syndrome. Int J Burns Trauma.

[76] Godoy DA, Di Napoli M, Rabinstein AA (2018). Cerebral fat embolism: recognition, complications, and prognosis. Neurocrit Care.

[77] Xu X-L, Xu P, Zheng R-Q (2016). A case of cerebral fat embolism. Chin Med J (Engl).

[78] Decaminada N, Thaler M, Holler R (2012). Brain fat embolism. A report of two cases and a brief review of neuroimaging findings. Neuroradiol J.

[79] Chiappa V, Gonzalez RG, Manian FA, Deshpande V (2016). Case 23–2016: a 46-year-old man with somnolence after orthopedic surgery. N Engl J Med.

[80] Goenka N, Ropper AH (2012). Cerebral fat embolism. N Engl J Med.

[81] Aravapalli A, Fox J, Lazaridis C (2009). Cerebral fat embolism and the “starfield” pattern: a case report. Cases J.

[82] Herway ST, Slotto J, Harlan E, Newhouse B (2016). Cerebral fat embolism syndrome. Anesthesiology.

[83] Duran L, Kayhan S, Kati C (2014). Cerebral fat embolism syndrome after long bone fracture due to gunshot injury. Indian J Crit Care Med.

[84] Al-Shaer DS, Ayoub O, Ahamed NA (2016). Cerebral fat embolism syndrome following total knee replacement causing a devastating neurocognitive sequelae. Neurosciences.

[85] Rughani AI, Florman JE, Seder DB (2011). Clinical and radiographic improvement following cerebral fat emboli. Neurocrit Care.

[86] Manousakis G, Han DY, Backonja M (2012). Cognitive outcome of cerebral fat embolism. J Stroke Cerebrovasc Dis.

[87] Forteza AM, Rabinstein A, Koch S (2002). Endovascular closure of a patent foramen ovale in the fat embolism syndrome: changes in the embolic patterns as detected by transcranial Doppler. Arch Neurol.

[88] Carlson DS, Pfadt E (2011). Fat embolism syndrome. Nursing (Lond).

[89] Kumar S, Gupta V, Aggarwal S (2012). Fat embolism syndrome mimicker of diffuse axonal injury on magnetic resonance imaging. Neurol India.

[90] Caricato A, Russo G, Biasucci DG, Annetta MG (2017). Fat embolism syndrome. Intensive Care Med.

[91] Uransilp N, Muengtaweepongsa S, Chanalithichai N, Tammachote N (2018) Fat embolism syndrome: a case report and review literature. Case Rep. Med 10.1155/2018/1479850 PMC5949181 29853905

[92] Shaikh N, Parchani A, Bhat V, Kattren MA (2008). Fat embolism syndrome: clinical and imaging considerations: case report and review of literature. Indian J Crit Care Med.

[93] Lin K-Y, Wang K-C, Chen Y-L (2015). Favorable outcome of cerebral fat embolism syndrome with a glasgow coma scale of 3: a case report and review of the literature. Indian J Surg.

[94] Whalen LD, Khot SP, Standage SW (2014). High-dose rosuvastatin treatment for multifocal stroke in trauma-induced cerebral fat embolism syndrome: a case report. PediatrNeurol.

[95] Chen PC, Hsu CW, Liao WI (2013). Hyperacute cerebral fat embolism in a patient with femoral shaft fracture. Am J Emerg Med.

[96] May J, Sullivan JC, LaVie D (2016). Inside out: bone marrow necrosis and fat embolism complicating sickle-β+ thalassemia. Am J Med.

[97] Marshall GB, Heale VR, Herx L (2004). Magnetic resonance diffusion weighted imaging in cerebral fat embolism. Can J NeurolSci.

[98] Sethi D, Kajal S, Saxena A (2015). Neuroimaging findings in a case of cerebral fat embolism syndrome with delayed recovery. Indian J Crit Care Med.

[99] Yeo SH (2013). Pulmonary and cerebral fat embolism syndrome after total knee replacement. J Clin Med Res.

[100] Shacklock E, Gemmell A, Hollister N (2017). Neurological effects of fat embolism syndrome: a case report. J Intensive Care Soc.

[101] You JS, Kim SW, Lee HS, Chung SP (2010). Use of diffusion-weighted MRI in the emergency department for unconscious trauma patients with negative brain CT. Emerg Med J.

[102] Nastanski F, Gordon WI, Lekawa ME (2005). Posttraumatic paradoxical fat embolism to the brain: a case report. J Trauma Inj Infect Crit Care.

[103] Han Y-T, Tang J, Gao Z-Q, Hu H-T (2016). Clinical features and neuroimaging findings in patients with cerebral fat embolism. Chin Med J (Engl).

[104] Ryu CW, Lee DH, Kim TK (2005). Cerebral fat embolism: diffusion-weighted magnetic resonance imaging findings. ActaRadiol.

[105] Sasano N, Ishida S, Tetsu S (2004). Cerebral fat embolism diagnosed by magnetic resonance imaging at one, eight, and 50 days after hip arthroplasty: a case report. Can J Anaesth.

